# The Effect of Kelulut Honey on Fasting Blood Glucose and Metabolic Parameters in Patients with Impaired Fasting Glucose

**DOI:** 10.1155/2019/3176018

**Published:** 2019-02-03

**Authors:** Mohd Radzniwan Rashid, Khairun Nain Nor Aripin, Fathima Begum Syed Mohideen, Nizam Baharom, Khairani Omar, Nik Mohd Shafikudin Md Taujuddin, Hazira Hanum Mohd Yusof, Faizul Helmi Addnan

**Affiliations:** ^1^Faculty of Medicine and Health Science, Universiti Sains Islam Malaysia, Pandan Indah, Malaysia; ^2^Faculty of Medicine, Universiti Kebangsaan Malaysia, Kuala Lumpur, Malaysia; ^3^University Health Centre, Universiti Sains Islam Malaysia, Bandar Baru Nilai, Malaysia; ^4^Klinik Kesihatan Kampung Pandan, Kuala Lumpur, Malaysia

## Abstract

**Background:**

Impaired fasting glucose (IFG) poses a higher risk of diabetes. Honey has been reported to improve metabolic abnormalities including lowering hyperglycemia. This study is sought at determining the effect of Malaysian Kelulut honey (KH) on fasting glucose levels and metabolic parameters in IFG patients.

**Methods:**

This quasi-experimental intervention study of 30-day duration was conducted among 60 adult patients with IFG. They were allocated into taking 30 g/day KH group (experimental group, *n*=30) and not taking KH group (control group, *n*=30). Body mass index (BMI), waist circumference, blood pressure (BP), fasting glucose, and lipid profile levels (total cholesterol, triglyceride, high-density lipoprotein, and low-density lipoprotein) were measured before and after treatment.

**Results:**

There was no significant difference in all measured variables at day 30 compared to day 1 within both groups. Similarly, all measured variables neither at day 1 nor at day 30 had shown a statistically significant difference between the groups.

**Conclusions:**

Daily intake of 30 g KH for 30 days resulted in insignificant effect on fasting glucose, fasting lipid profiles, and other metabolic parameters in patients with IFG. Further studies that employ longer study duration are needed to ascertain the finding.

## 1. Introduction

Impaired fasting glucose (IFG) is a prediabetes condition along with impaired glucose tolerance (IGT). Both conditions pose a substantial increased risk of cardiovascular disease. In addition, they are associated with metabolic syndrome which put them at higher risk for coronary artery disease [1] and death; hence, early detection and intervention of prediabetes condition is beneficial [2]. Nonetheless, patients are often asymptomatic at this stage and can only be diagnosed through the oral glucose tolerance test (OGTT). The third NHMS in 2006 in Malaysia found that the combined prevalence of impaired fasting glycaemia and impaired glucose tolerance was 4.2% [3]. In other parts of the world, the population-based study amongst Iranian urban residents had reported the prevalence of IFG was 8.7% in men and 6.3% in women [4], whereas the Diabetes Screening in Canada (DIASCAN) study had documented the prevalence of IFG was 2.5% [5].

Patients with IFG have a significant risk of developing diabetes mellitus (DM) [6] in which the proportion that developed into frank DM varies widely. For instance, the Hoorn study found that 33% of patients with IFG alone developed DM within 5–8 years [7]. The Paris Prospective Study reported that much lower proportions of 2.7% isolated IFG would develop DM over 2.5 years of follow-up [8]. An Italian study spanning 11.5 years found that 9.1% of patients with isolated IFG developed DM [9]. Consequently, early screening programmes are becoming more crucial. Clinicians are advised to target patients with risk factors for prediabetes condition such as family history of DM, body mass index greater than 25 kg/m^2^, sedentary lifestyle, hypertension, dyslipidaemia, history of gestational diabetes or history of having macrosomic baby, and polycystic ovary syndrome [10].

Up until now, there is strong evidence that lifestyle changes and/or pharmacotherapy such as metformin are effective in reducing the progression of prediabetes into frank DM [11]. These lifestyles changes include modest weight loss, good dietary habits, and regular physical activity. However, lifestyle interventions can be difficult to implement because it is impractical for the health care team to provide intensive dietary and exercise interventions similar to those used in clinical trials. Hence, many patients resort to alternative ways to prevent getting DM. This includes the use of herbal preparations, dietary components or supplements, and other natural products such as honey [12]. In the recent years, there has been an increased interest in the therapeutic uses of honey because it has demonstrated several health benefits in managing chronic conditions including DM [13].

Honey is an organic substance produced by bees from nectar. It contains different compounds such as carbohydrates, conventional minerals, proteins, vitamins, organic acids, enzymes and antioxidants such as catalase and peroxidase, alkaloids, polyphenols, and flavonoids [14–16]. In addition, it has been shown to scavenge reactive oxygen species, ameliorate oxidative stress, and reduce hyperglycaemia [17, 18]. Increased generation of reactive oxygen species (ROS) resulting from metabolism of excessive glucose and/or free fatty acids has been identified as a contributor to the deterioration of pancreatic *β*-cell function. Honey could impede this mechanism as it has antioxidative properties.

In Malaysia, available honey includes Kelulut, Tualang, Gelam, Acacia, and Pineapple among others. Kelulut honey (KH) is produced by stingless bees from Trigona spp. In comparison with other honey, KH is more diluted and has special sour-like taste and smell [19]. KH has been consumed traditionally in Malaysia with beliefs on its antiageing effect, enhancing libido and immune system, killing bacteria, treating bronchial phlegm, and relieving sore throat, cough, and cold [20]. In addition, several studies had shown that it is also proven to possess various pharmacological properties such as anti-inflammatory [21], antioxidant [22], and antibacterial [23] properties. In an animal study, KH did not cause adverse side effects nor did it cause abnormal values of blood profile, liver enzymes, and kidney function [24].

Similar to many communities around the world, honey in Malaysia is often treated as alternative medicine for cure and illness prevention. Nonetheless, evidence is lacking especially from human studies in eliciting the beneficial effects on patients with impaired fasting glucose. Therefore, this study aimed to find whether KH could benefit patients with impaired fasting glucose on several metabolic parameters, namely, glucose control, patients' weight, waist circumference, cholesterol, and blood pressure.

## 2. Materials and Methods

### 2.1. Study Design and Intervention

This is a 30-day quasi-experimental intervention study to evaluate the effects of Malaysian Kelulut honey on fasting plasma glucose (FPG), fasting lipid profiles, and body weight among participants in two centres, namely, Universiti Sains Islam Malaysia (USIM) Specialist Centre, Negeri Sembilan, Malaysia, and Faculty of Medicine and Health Sciences, Kuala Lumpur in Malaysia. The data were collected from September 2017 to January 2018. The ethical approval to carry out this study was obtained from the Research Ethic Committee of Universiti Sains Islam Malaysia (Project code: PPP-FPSK-15515-00). All participants were informed verbally of the study requirements and gave written informed consent before enrolment.

### 2.2. Sample Size Calculation

PS2 software (mean difference of 29, standard deviation of 37.5, confidence interval of 95%, power of 80%, and 20% dropouts) was used to calculate the sample size. Minimum sample size based on 1 : 1 (case-to-control ratio) was 30 participants in each arm. Hence, there were a total of 60 IFG participants. They were allocated to either the intervention group (*n*=30) or a control group (*n*=30).

### 2.3. Participants

Participants diagnosed as having impaired fasting glucose (IFG) and aged more than 18 years were included. IFG is defined as fasting plasma glucose values of 6.1 to 6.9 mmol/L [25]. Participants who were taking honey and any form of the herbal extract in the last 3 months before study entry, pregnant women, and those who had history of drug or alcohol abuse were excluded.

Participants in the experimental group received 30 g/day of KH, while participants in the control group did not receive any honey. The honey was procured from the Bayu Kelulut® company, based in the Kedah state, Malaysia, and their product is certified by Malaysian Good Manufacturing Practices and Malaysian Agricultural Research and Development Institute (MARDI).

Participants in both groups underwent their normal consultation from the physicians, and they were not advised to any special diet regimen. The participants were followed up, and any side effects on consuming KH were recorded. Any new symptom that appeared after commencement of intervention was considered a side effect and recorded. At the end of the study, 3 participants from each arm were lost to follow-up due to logistic reasons (3 participants), loss of interest (1 participant), and unspecified reasons (2 participants).

### 2.4. Data Collection

Weight, height, waist circumference, and blood pressure were measured before and after intervention using a standard protocol. Height and body weight were measured with the participants dressed in light clothing after an overnight fast. The body weight of each subject was measured with a standard scale to an accuracy of ±0.1 kg, and height was measured to an accuracy of ±0.1 cm. The body mass index (BMI) was calculated as weight (kg) divided by height squared (m^2^). Blood samples for biochemical analysis were collected in the morning after an overnight fast from each subject at day 1 and day 30.

### 2.5. Biochemical Analysis

The blood was sent to an accredited private laboratory and was analysed by the enzymatic method using an Abbott Architect (United Kingdom) chemical analyser. Fasting blood glucose (FBG) and lipid profile comprising total cholesterol (TC), high-density lipoprotein (HDL), low-density lipoprotein (LDL), and triglyceride (TG) were measured for each participant.

### 2.6. Statistical Analysis

The Kolmogorov–Smirnov test and Shapiro–Wilk test were used to evaluate normal distribution. Data were presented as either mean ± standard deviation or median with an interquartile range. The Student independent *t*-test, Mann–Whitney *U*-test, and chi-square test were used appropriately for comparison of variables between intervention and control groups. The Student paired *t*-test and Wilcoxon signed-ranks test were used appropriately for comparison of variables between pre- and postintervention. All analyses were conducted by using SPSS version 21, with a significance level set <0.05.

## 3. Results

Thirty participants were enrolled for the intervention group with KH, while another thirty were enrolled as the control group. The mean age, proportions of gender distribution, ethnicity, marital status, occupation category, education levels, and income levels were not statistically significant between the intervention (KH) and control groups (Table 1).

Table 2 describes the clinical parameters at baseline of both the KH and control groups. Again, there was no significant difference between the two groups as shown by bivariate analysis. The approximate duration of being diagnosed with impaired fasting glucose was less than 3 years. The mean fasting blood glucose level for both groups was 6.2 mmol/L. Both groups also demonstrated similar lipid profiles at baseline with the total cholesterol around 5.3 mmol/L.

Mean differences in postintervention outcomes were compared between KH and control groups (Table 3). The control group demonstrated a mean difference of 0.13 ± 0.69 mmol/L for the fasting blood glucose level, while the mean difference for the KH was negligible. However, there was no significant difference for the changes between the groups. Similarly, there was no significant difference between the groups for changes in lipid profiles, BMI, and blood pressure readings.

Consumption of Kelulut honey for 30 days did not affect levels of fasting blood glucose, lipid profiles, BMI, and blood pressure readings (Table 4). Likewise, similar findings were shown in the control group (Table 5).

## 4. Discussion

In our study, we hypothesised that consumption of KH for 30 days may result in an improvement of fasting blood glucose and other metabolic parameters in patients with IFG. In this study, KH was considered as part of the daily diet for the participants. In the present study, we administered the honey at 30 g/day to the participants.

Thirty grams of KH contain on average 21 g of carbohydrate [26] which yields 84 kcal. This amount of carbohydrate (simple sugars) administered to our participants is within tolerable intake as stated in the Malaysian Dietary Guideline 2017 [27]. The dosage used was within the tolerable amount without any documented side effect as demonstrated in other studies [28, 29]. An *in vivo* study has shown that KH did not cause toxicity effect when administered at a concentration of 1.18 g/kg of body weight [24]. Our present study used a much lower concentration than this.

The participants were not required to record in detail their dietary pattern and physical activity during the study. However, they were advised to retain their typical diet and physical activities during the intervention period. This enabled them to practice their routine activities genuinely without adding any anxiety effect or stress. Practically, this is good as this may reflect truly their body response towards honey.

The approximate duration of IFG noted in our study was less than 3 years. The relatively short duration of this condition may be advantageous for honey-supplement therapy as to revert the prediabetes state. The mean values of fasting blood glucose (FBG) in the controlled and the intervention groups observed in this study were within the definition of IFG which is currently practiced in Malaysia [25].

At the end of the intervention period, we observed that, within both groups, there was no significant difference in FBG. To the best of our knowledge, we did not find any similar study that tested the effect of stingless bee honey for those with IFG. Our finding was in agreement with the study of Al Waili [30] who documented a nonsignificant difference in FBG in healthy subjects that consumed honey. Nonetheless, an 8-week study among normal individuals by Bahrami et al. [13] found that there was a significant reduction in FBG among those taking honey. In contrast, Abdulrhman et al. [28] have shown that consumption of honey for 12 weeks by type 1 diabetic patients resulted in significant reduction of FBG. It was demonstrated that honey consumption causes significant elevation of C-peptide levels in type 1 diabetic and healthy subjects [31]. The plasma concentrations of C-peptide effectively reflect the endogenous insulin secretion. C-peptide is part of the proinsulin molecule, and it is cosecreted when insulin is released from the proinsulin molecule [32]. The ability of honey to stimulate the *β*-cells of the pancreas confers promising effect towards lowering blood glucose levels. However, in the present study, we could not observe this effect.

We believe that the 30-day period of honey consumption was not sufficient to observe significant changes in the FBG level. Another factor to consider is that our subjects have lower FBG; hence, it is difficult to observe any significant changes. Discrepancies of the results could also be due to the type of honey used as the nutritional values of the honey are significantly dependent on different botanical sources and geographical locations (soil and climate conditions) [33].

As for the blood lipid profiles, we did not observe any significant changes in the level of TC, TG, LDL, and HDL in both groups at the end of the study. Our findings were in agreement with the study of Munstedt et al. [34] which found no significant changes in blood lipid profiles in patients with hypercholesterolaemia receiving honey. This is in contrast with other studies which showed natural honey decreases TC and LDL and increases HDL in normal subjects [30], overweight or obese subjects [35], and type 1 diabetic patients [28]. It is speculated that the antioxidant properties of honey can act as hypolipidemic effects. Unfortunately, this lowering cholesterol effect of honey was not observed in our study.

Our study shows that honey consumption did not cause an increase of body weight, although honey is rich in sugar content. Importantly, decreased body weight was observed when honey was consumed by diabetic patients [13]. These observations make honey a suitable food to be included in the daily diet. In addition, our study also shows that honey consumption did not have an effect on blood pressure. Contrarily, other studies have reported that honey has antihypertensive property when administered to hypertensive rats [36] and hypertensive patients [37].

Some limitations were observed in our study. These include predominant participants of Malay ethnicity, short duration of intervention, and unrecorded food and calories intake and physical activities. During the study period, all the participants have received nutritional advice about maintaining an isocaloric diet; however, we were unable to verify the participants adhered to the advice given. Longer study duration and proper documentation on their diet and physical activities are suggested for future studies. HbA1c is also recommended to be used as a marker of blood glucose control as it accurately measures the glucose control. Some participants informally responded that they felt better after taking the honey. This could be possibly due to placebo effects or nutraceutical effects of taking honey.

Nonetheless, our study is the first study to investigate the effect of Malaysian honey consumption among impaired fasting glucose patients. In conclusion, KH consumption for 30 days does not have an effect on fasting blood glucose, fasting lipid profiles, and other metabolic parameters in patients with IFG.

## Figures and Tables

**Table 1:** Sociodemographic profiles of the participants with impaired fasting glucose in a quasi-experimental intervention study.

Variables	Kelulut honey	Control	All
Age (mean ± SD)	52.8 ± 10.4	50.4 ± 12.5	51.6 ± 11.5
*Gender*			
Male	17 (56.7)	13 (43.3)	30 (50.0)
Female	13 (43.3)	17 (56.7)	30 (50.0)
*Ethnicity*			
Malay	25 (83.3)	20 (66.6)	45 (75.0)
Non-Malay	5 (16.6)	10 (33.3)	15 (25.0)
*Marital status*			
Married	29 (96.7)	26 (86.7)	55 (91.7)
Unmarried	1 (3.3)	4 (13.3)	5 (8.3)
*Occupation*			
Working	18 (60)	23 (76.7)	41 (68.3)
Not working	12 (40)	7 (23.3)	19 (31.7)
*Education*			
Lower	19 (63.3)	22 (73.3)	41 (68.3)
Higher	11 (36.6)	8 (26.7)	19 (31.7)
*Income level*			
Low	13 (43.3)	15 (50)	28 (46.7)
Medium or high	17 (56.6)	15 (50)	32 (53.3)

**Table 2:** Baseline outcomes of participants with impaired fasting glucose in a quasi-experimental intervention study.

	Kelulut honey	Control	All
Duration of IFG (years)	1.6 ± 1.3	1.0 ± 1.2	1.3 ± 1.3
*Hypertension*			
Yes	11 (36.7)	12 (40)	23 (38.3)
No	19 (63.3)	18 (60)	37 (61.7)
*Hypercholesterolaemia*			
Yes	22 (73.3)	25 (83.3)	47 (78.3)
No	8 (26.6)	5 (16.7)	13 (21.7)
Body mass index	29.5 ± 4.8	29.8 ± 5.6	29.7 ± 5.2
Fasting blood glucose (mmol/L)	6.2 ± 1.4	6.2 ± 1.0	6.2 ± 1.2
Total cholesterol (mmol/L)	5.3 ± 1.2	5.2 ± 0.9	5.3 ± 1.0
High-density lipoprotein (mmol/L)	1.2 ± 0.3	1.3 ± 0.3	1.3 ± 1.2
Low-density lipoprotein (mmol/L)	3.4 ± 1.3	3.3 ± 0.8	3.4 ± 1.1
Triglycerides^*∗*^ (mmol/L)	1.5 (1.1)	1.4 (0.9)	1.4 (1.1)
Systolic blood pressure (mmHg)	134.9 ± 14.6	133.8 ± 12.1	134.9 ± 13.1
Diastolic blood pressure (mmHg)	86.3 ± 9.4	83.0 ± 9.7	85.1 ± 9.5

Values are mean ± SD of observed findings. ^*∗*^Median (IQR).

**Table 3:** Analysis of mean difference in postintervention outcomes of participants with impaired fasting glucose in a quasi-experimental intervention study.

	Group	Mean ± SD	*t*-test	*p* value (*t*-test)
Fasting blood glucose (mmol/L)	Kelulut honey	0.00 ± 0.90	−0.592	0.556
	Control	0.13 ± 0.69		

Total cholesterol (mmol/L)	Kelulut honey	0.08 ± 0.64	1.145	0.257
	Control	−0.11 ± 0.56		

High-density lipoprotein (mmol/L)	Kelulut honey	−0.06 ± 0.23	−0.807	0.423
	Control	−0.01 ± 0.23		

Low-density lipoprotein (mmol/L)	Kelulut honey	−0.10 ± 1.07	0.637	0.527
	Control	−0.25 ± 0.69		

Triglycerides (mmol/L)	Kelulut honey	0.00 ± 0.57^*∗*^	Na	0.909^*∗∗*^
	Control	0.00 ± 0.71^*∗*^		

Body mass index (kg/m^2^)	Kelulut honey	0.62 ± 0.85	0.299	0.796
	Control	0.56 ± 0.56		

Systolic blood pressure (mm/Hg)	Kelulut honey	1.07 ± 6.09	1.921	0.060
	Control	−3.11 ± 9.60		

Diastolic blood pressure (mm/Hg)	Kelulut honey	0.15 ± 7.59	0.040	0.969
	Control	−0.07 ± 6.77		

^*∗*^Median ± IQR. ^*∗∗*^Mann–Whitney *U*-test.

**Table 4:** Pre- and postintervention outcomes in participants with impaired fasting glucose who consumed 30 g Kelulut honey for 30 days in a quasi-experimental intervention study.

	Mean ± SD	*t*-test	*p* value (paired *t*-test)
*Fasting blood glucose (mmol/L)*			
Pre	6.33 ± 1.36	0.469	0.643
Post	6.33 ± 1.14		
*Total cholesterol (mmol/L)*			
Pre	5.34 ± 1.15	−0.616	0.543
Post	5.41 ± 1.11		
*High-density lipoprotein (mmol/L)*			
Pre	1.25 ± 0.28	1.329	0.195
Post	1.19 ± 0.24		
*Low-density lipoprotein (mmol/L)*			
Pre	3.47 ± 1.30	0.469	0.643
Post	3.37 ± 1.19		
*Triglycerides (mmol/L)*			
Pre	1.51 ± 1.10^*∗*^	−0.046	0.964^*∗∗*^
Post	1.56 ± 1.40^*∗*^		
*Body mass index (kg/m* ^*2*^)			
Pre	29.32 ± 4.80	−0.819	0.420
Post	29.39 ± 4.81		
*Systolic blood pressure (mm/Hg)*			
Pre	136.04 ± 14.48	−0.916	0.368
Post	137.11 ± 13.32		
*Diastolic blood pressure (mm/Hg)*			
Pre	87.00 ± 8.91	−0.101	0.920
Post	87.15 ± 10.84		

^*∗*^Median ± IQR. ^*∗∗*^Wilcoxon signed-ranks test.

**Table 5:** Pre- and postintervention outcomes in participants with impaired fasting glucose in the control group in a quasi-experimental intervention study.

	Mean ± SD	*t*-test	*p* value (paired *t*-test)
*Fasting blood glucose (mmol/L)*			
Pre	6.26 ± 0.99	−0.988	0.332
Post	6.39 ± 1.08		
*Total cholesterol (mmol/L)*			
Pre	5.24 ± 0.95	1.039	0.308
Post	5.13 ± 1.03		
*High-density lipoprotein (mmol/L)*			
Pre	1.27 ± 0.26	0.197	0.845
Post	1.26 ± 0.27		
*Low-density lipoprotein (mmol/L)*			
Pre	3.38 ± 0.84	1.922	0.065
Post	3.13 ± 1.07		
*Triglycerides (mmol/L)*			
Pre	1.35 ± 0.88^*∗*^	−0.411	0.681^*∗∗*^
Post	1.35 ± 1.26^*∗*^		
*Body mass index (kg/m* ^*2*^)			
Pre	29.42 ± 5.38	−1.418	0.167
Post	29.55 ± 5.39		
*Systolic blood pressure (mm/Hg)*			
Pre	134.12 ± 12.50	1.713	0.098
Post	131.00 ± 13.30		
*Diastolic blood pressure (mm/Hg)*			
Pre	83.07 ± 9.67	−0.056	0.959
Post	83.14 ± 12.05		

^*∗*^Median ± IQR. ^*∗∗*^Wilcoxon signed-ranks test.

## Data Availability

All the data obtained during the study are kept in a secured research file to ensure confidentiality.
